# Efficacy and safety of single-trajectory posterior subthalamic area and subthalamic nucleus deep brain stimulation for dystonic tremor: a retrospective pilot study

**DOI:** 10.1007/s00415-025-13569-0

**Published:** 2026-01-06

**Authors:** Jianyi Shen, Zhengyu Lin, Suzhen Lin, Peng Huang, Yixin Pan, Bomin Sun, Jianqing Ding, Yiwen Wu, Dianyou Li

**Affiliations:** 1 Department of Neurosurgery, Center for Functional Neurosurgery, Ruijin Hospital, affiliated with Shanghai Jiaotong University School of Medicine, 197 Ruijin Er Road, Shanghai, 200025 China; 2 Institute of Aging & Tissue Regeneration, Renji Hospital, Shanghai Jiao Tong University School of Medicine, Shanghai, China; 3 Department of Neurology & Institute of Neurology, Ruijin Hospital, affiliated with Shanghai Jiaotong University School of Medicine, 197 Ruijin Er Road, Shanghai, 200025 China

**Keywords:** Posterior subthalamic area, Subthalamic nucleus, Deep brain stimulation, Dystonic tremor

## Abstract

**Objective:**

To evaluate the feasibility, safety, and preliminary efficacy of a novel single-lead, dual-target deep brain stimulation (DBS) approach targeting the posterior subthalamic area (PSA) and subthalamic nucleus (STN) for dystonic tremor.

**Methods:**

This retrospective pilot study reviewed outcomes of six consecutive patients with medically refractory dystonic tremor who underwent single-lead PSA-STN DBS at our center (June–December 2024). Clinical outcomes were assessed using the BFMDRS and FTMTRS scales. A formal blinded crossover assessment was performed in three patients to compare PSA-only, STN-only, and combined stimulation. Chronic settings were selected via patient-directed optimization.

**Results:**

All six patients completed follow-up (100% retention) and achieved stable chronic stimulation programs. Five patients (83.3%) independently selected combined PSA + STN stimulation; one preferred STN-only. At LFU (6–12 months postoperatively), the mean BFMDRS-Motor score decreased by 78.1% and FTMTRS by 87.1%. The crossover assessment (*n* = 3) showed that combined stimulation outperformed single-target stimulation. No serious adverse events occurred. All efficacy analyses are exploratory.

**Conclusion:**

This single-lead, dual-target PSA-STN DBS approach demonstrates feasibility and preliminary efficacy for dystonic tremor. Prospective controlled trials are warranted.

**Supplementary Information:**

The online version contains supplementary material available at 10.1007/s00415-025-13569-0.

## Introduction

Dystonia with prominent tremor (hereafter referred to as dystonic tremor [DT]) is a complex hyperkinetic phenotype in which dystonia—sustained or intermittent muscle contractions causing abnormal movements and postures—is accompanied by a prominent, often rhythmic tremor in the same or overlapping body part. Recent MDS updates to dystonia definitions recognize that dystonic movements and postures may have a tremulous quality and emphasize careful phenomenological description when tremor coexists with dystonia [1]. While recent expert commentaries encourage more descriptive phenomenological classification of tremor in the context of dystonia, we retain the term dystonic tremor to denote the clinical phenotype of our cohort—patients with isolated dystonia presenting with a prominent, often rhythmic tremor component—consistent with established usage in the Deep brain stimulation (DBS) literature [2–4].

DBS has emerged as the most effective surgical treatment for medically refractory dystonia [2, 3]. Both the globus pallidus internus (GPi) and subthalamic nucleus (STN) have been established as effective targets for treating dystonia [4]. Our center has extensive experience with STN and GPi DBS for dystonia, and we previously published a comprehensive comparison of STN and GPi efficacy in treating isolated dystonia. We demonstrated that the GPi and STN are safe and effective targets for isolated dystonia [5, 6].

However, while both STN and GPi stimulation can effectively control dystonic symptoms in DT patients, their efficacy in controlling the tremor component remains variable and inconsistent across patients [4, 7]. The ventralis intermedius nucleus (VIM) of the thalamus is commonly targeted for tremor control, and combined GPi and VIM DBS treatment has shown superior outcomes compared to single-target stimulation [8, 9]. Nevertheless, this dual-target approach requires the implantation of two separate DBS devices, increasing invasiveness and cost.

The posterior subthalamic area (PSA) has emerged as a promising target for tremor control in recent years [10–12]. In our 2024 publication, we demonstrated that PSA-DBS is comparable with VIM-DBS in suppressing tremors, superior in improving disease-specific quality of life, and possibly more effective in reducing head tremor [13]. Importantly, the anatomical proximity of the PSA and STN presents a unique opportunity: the possibility of targeting both structures with a single electrode trajectory. Therefore, we explored this alternative combined targeting strategy as a potential solution for DT. This single-electrode, dual-target approach could potentially address both dystonic and tremor components of DT while avoiding the increased surgical trauma and costs associated with implanting two separate DBS systems. Here, we present a retrospective analysis of our initial clinical experience with PSA-STN DBS in six DT patients, including a blinded crossover assessment in three patients to objectively evaluate the individual and combined effects of each target.

## Patients and methods

### Study design

This retrospective pilot study was designed to assess the feasibility, safety and preliminary clinical outcomes of a novel dual-target DBS approach. Consistent with pilot study methodology[14, 15], our primary objectives were to evaluate: (1) procedural feasibility, (2) safety, (3) patient acceptability, and (4) retention and protocol completion. Efficacy analyses are exploratory and hypothesis-generating, intended to inform future confirmatory trials.

### Patients

We conducted a retrospective analysis of the clinical outcomes of six patients with medically refractory isolated dystonia and presented with a prominent rhythmic tremor phenotype in the affected body region who underwent single-lead, dual-target PSA-STN DBS surgery at our center between June 2024 and December 2024. Six patients comprised the complete cohort during this period; no eligible patient was excluded. The date of surgery and follow-up duration for each patient are provided in Table 1. This retrospective pilot study was approved by the Institutional Review Board of Ruijin Hospital (Approval ID: Clinical Ethics Review (2024) No. 84; Approval Date: April 11, 2024). All patients provided written informed consent for the DBS procedure and for the use of their anonymized clinical data for research purposes. The study adhered to the principles of the Declaration of Helsinki. A copy of the IRB approval is available upon request.

**Table 1:** Demographic and clinical characteristics

Patient	Sex	Family history	Age at onset (year)	Age at surgery (year)	Surgery date (month/year)	Duration of symptoms (month)	Dystonia distribution	Tremor site	LFU (month)	Follow-up	BFMDRS-Motor score		BFMDRS-Disability score	FTMTRS
Patient 1	F	None	50	65	September 2024	190	Blepharospasm; mouth; torticollis;upper limb dystonia	arms; head; voice; face	6	Baseline		26	16	25
										LFU		8	6	6
Patient 2	F	None	49	64	June 2024	189	Blepharospasm; mouth; torticollis/laterocollis	head; voice; face	12	Baseline		13.5	8	11
										LFU		2	4	0
Patient 3	M	None	53	59	June 2024	72	Blepharospasm; mouth; torticollis/retrocollis;truncal dystonia	head; face; arms	12	Baseline		15	6	18
										LFU		2	1	0
Patient 4	M	None	57	65	July 2024	107	Blepharospasm; laryngeal dystonia; anterocollis	head; voice; face	12	Baseline		13	6	13
										LFU		3	2	2
Patient 5	F	None	45	61	August 2024	199	Writer's cramp; torticollis; truncal dystonia	head; voice; arms	12	Baseline		20	11	17
										LFU		2	1	1
Patient 6	F	None	24	33	September 2024	104	Torticollis; upper limb dystonia	head	6	Baseline		13	12	9
										LFU		5	2	3
Mean ± SD			46.3 ± 11.7	57.8 ± 12.4		143.5 ± 55.4			10 ± 3.1	Baseline		16.8 ± 5.3	9.8 ± 3.9	15.5 ± 5.8
										LFU		3.7 ± 2.4	2.8 ± 2.0	2 ± 2.3
					P-value^a^ (Baseline vs. LFU)							0.0006(0.0312)	0.0025(0.0312)	0.0012(0.0312)

*LFU* last follow up, All patients had isolated dystonia; secondary causes were excluded by MRI and comprehensive laboratory screening

^a^*P*-values were calculated using the paired Student's t-test, with results from the Wilcoxon signed-rank test shown in parentheses

Inclusion criteria were: (i) a diagnosis of isolated dystonia and presented with a prominent rhythmic tremor phenotype in the affected body region according to MDS consensus criteria [1].The dystonia was the primary and most disabling feature, or the tremor was refractory to medications typically used for essential tremor. (ii) no acquired etiology or neurodegenerative diseases or preoperative brain MRI with any signs of structural brain damage. (iii) accurate electrode position confirmed by pre- and post-operative neuroimaging. (iv) the patient and/or their caregiver needed to demonstrate the ability and willingness to manage the patient programmer to switch between stimulation programs at home.

Patients were excluded if they had severe cognitive impairment or an uncontrolled psychiatric illness that would interfere with informed consent or postoperative management. Exclusion criteria also included the presence of any structural brain anomalies on preoperative MRI that could be responsible for the symptoms or any general contraindications to neurosurgery.

Etiologic evaluation included brain MRI and comprehensive laboratory screening (copper metabolism, thyroid function, vitamin levels, etc.) to exclude secondary causes; no structural lesions or neurodegenerative features were identified. Clinical characteristics, including age at onset, distribution, and family history, are detailed in Table 1. Genetic testing was not performed.

The cohort comprised four women and two men who met MDS consensus criteria. Patient 1 developed limb tremor and cramping that advanced to facial dystonia, torticollis and laryngeal tremor. Patient 2 presented with cervical dystonia (torticollis to the left with laterocollis to the right) that gradually progressed with marked head tremor, facial dystonia, and voice tremor. Patient 3 started with truncal dystonia that spread upward to torticollis–retrocollis and was joined by jerky head tremor plus facial and arm tremor. Patient 4 started with blepharospasm and anterocollis, later adding head tremor and laryngeal dystonia. Patient 5 had writer’s cramp that extended to torticollis with tremor and voice involvement. Patient 6 initially noted mild right torticollis, and eight years later the dystonic head turn and superimposed tremor intensified and postural arm tremor emerged. All had trialed medications and/or botulinum toxin without sustained benefit.

### Surgical procedures

The PSA and STN were simultaneously targeted using a single DBS trajectory, as described in our previous work [16]. In contrast to our routine STN-DBS procedure for dystonia, in which the dorsolateral sensorimotor STN serves as the primary target and the trajectory is optimized to maximize intranuclear STN coverage, the present approach designates the PSA as the primary target. The PSA was directly visualized on axial T2-weighted MRI as a hypointense region located medial to the posterior tail of the STN and lateral to the red nucleus, at the level of the maximal diameter of the red nucleus. The STN was similarly identified on T2-weighted images as the hypointense biconvex structure lateral to the red nucleus. The trajectory was planned using the PSA as the primary target; the coronal and sagittal angles were then adjusted to obtain a trajectory that also traversed the dorsal STN, while avoiding sulci, blood vessels, and ventricles. This strategy positions the ventral electrode contacts in the PSA and the dorsal contacts in the dorsal STN region. The Leksell stereotactic frame arc and ring angles for all trajectories are provided in Supplementary Table S1.

Patient 1 received bilateral four-contact cylindrical leads (SceneRay SR1200; contact length 1.5 mm; inter-contact spacing 0.5 mm; total array span 7.5 mm), analogous to Medtronic 3389. Patients 2–6 received bilateral four-contact cylindrical leads (SceneRay SR1210; contact length 1.5 mm; inter-contact spacing 1.5 mm; total array span 10.5 mm), analogous to Medtronic 3387. All patients received a rechargeable implantable pulse generator (SceneRay SR1101). The 1.5 mm spacing configuration (SR1210) was preferred for most patients because its longer electrode array span (10.5 mm vs. 7.5 mm) facilitates simultaneous coverage of both the PSA and region dorsal to the STN within a single trajectory, which is essential for the dual-target stimulation strategy. The latter model was adopted after the first case to provide a longer contact span and potentially finer control over the dual-target region. The placement of all leads was verified by merging the postoperative CT with the preoperative MR. All participants had at least one contact in the PSA and one in the STN.

### DBS programming, patient-directed optimization, and crossover assessment

Initial programming commenced approximately one month after DBS implantation and was performed by an experienced movement disorder neurologist. A systematic monopolar review was performed on each contact to map immediate therapeutic effects on tremor and to identify stimulation-induced side-effect thresholds.

Based on this initial mapping and our dual-target hypothesis, a flexible, patient-centric programming strategy was implemented. Patients were offered three distinct stimulation programs: (1) PSA stimulation; (2) STN stimulation; (3) interleaved PSA + STN stimulation. The specific contact configuration (single- or dual-cathode) within each target was individualized based on the monopolar review; detailed programming parameters for each patient are provided in Table 2. Patients were given autonomy to switch between programs at home and to adjust stimulation amplitude within predefined safe ranges. Patients were instructed to alternate between programs during the initial two-month optimization period, maintaining each program for at least one week to allow adequate assessment of therapeutic effects. At the two-month follow-up, patient preferences were formally recorded; non-preferred programs were subsequently deactivated to simplify device management. Five of six patients independently selected combined PSA + STN stimulation; Patient 3 preferred STN-only stimulation based on his symptom profile. Non-preferred programs were subsequently deactivated to simplify device management.

**Table 2:** Optimal stimulation parameters

Patient	Side	Parameters
Patient 1	Left	c + 8–9-, 50 μs, 160 Hz, 4.0 mA c + 11-, 60 μs, 160 Hz, 0.5 mA
	Right	c + 0–1-, 30 μs, 160 Hz, 4.0 mA c + 3-, 50 μs, 160 Hz, 0.25 mA
Patient 2	Left	c + 9-, 50 μs, 160 Hz, 2.5 V c + 10–11-, 60 μs, 160 Hz, 3.7 V
	Right	c + 1-, 50 μs, 160 Hz, 2.75 V c + 2–3-, 60 μs, 160 Hz, 3.95 V
Patient 3	Left	c + 10–11-, 50 μs, 160 Hz, 2.15 V c + 11-, 60 μs, 160 Hz, 3.45 V
	Right	c + 2–3-, 50 μs, 160 Hz, 2.45 V c + 3-, 60 μs, 160 Hz, 3.75 V
Patient 4	Left	c + 9-, 60 μs, 125 Hz, 1.7 V c + 10-, 60 μs, 125 Hz, 2.9 V
	Right	c + 0-, 60 μs, 125 Hz, 1.5 V c + 2–3-, 60 μs, 125 Hz, 2.7 V
Patient 5	Left	c + 9–10-, 50 μs, 145 Hz, 2.55 V c + 11-, 50 μs, 145 Hz, 3.5 V
	Right	c + 0–1-, 30 μs, 145 Hz, 2.35 V c + 3-, 50 μs, 145 Hz, 3.75 V
Patient 6	Left	c + 8-, 30 μs, 160 Hz, 3.45 V c + 10–11-, 50 μs, 160 Hz, 3.75 V
	Right	c + 0-, 30 μs, 160 Hz, 3.15 V c + 2–3-, 50 μs, 160 Hz, 3.85 V

The numbers 0 to 3 and 8 to 11 were assigned to the left and right DBS lead from the ventral-most to dorsal-most contacts

During the last follow-up visit, a formal blinded crossover assessment was conducted in three patients (Patients 2, 5, and 6; at 12 months for Patients 2 and 5, and at 6 months for Patient 6). The other three patients declined due to personal time constraints or concerns about the extended assessment duration. Four stimulation conditions were tested: Off-stimulation, PSA-only, STN-only, and combined PSA + STN. After an initial 30 min Off period, baseline assessments were performed. The order of the three active stimulation conditions was randomized. Each active condition was tested following a 30 min wash-in period; a 30 min washout (Off) period was interposed between each active condition. Both the patient and evaluating neurologist were blinded to the active stimulation settings. Clinical assessments (BFMDRS and FTMTRS) were performed under each of the four conditions, yielding 8 scale administrations per patient. The complete protocol required approximately 4 h. All six patients were invited to participate; three volunteered and completed the protocol without adverse events. This protocol was designed primarily to assess acute tremor responses; dystonia findings are exploratory given the limited washout duration.

### Outcome measures

Clinical outcome data were retrospectively collected from patient records. These records included assessments performed preoperatively and at follow-up visits in the outpatient setting, with the last available assessment for each patient defined as the last follow-up (LFU). The Burke Fahn Marsden Dystonia Rating Scale (BFMDRS) was used to rate the severity of dystonia. Changes in tremor severity and functional impact were assessed using the Fahn-Tolosa-Marin tremor rating scale (FTMTRS). Because this was a retrospective pilot cohort, early postoperative BFMDRS and FTMTRS assessments were not uniformly available for all six patients at standardized time points. Therefore, the primary efficacy evaluation focused on comparisons between baseline and the LFU to avoid potentially biased inferences from incomplete early datasets. Patients were regularly followed up at the outpatient department by a specialist knowledgeable of the patients’ condition. Adverse events were recorded.

### Statistical analysis [17, 18]

Analysis population: All six implanted patients were included in safety, feasibility, and exploratory efficacy analyses. There were no missing data for primary outcomes.

Chronic stimulation outcomes (*N* = 6): Clinical outcomes (BFMDRS-Motor, BFMDRS-Disability, FTMTRS) were assessed at baseline and last follow-up (LFU). Within-patient changes are summarized as means ± standard deviations and percentage improvements. Paired t-tests were used for within-patient comparisons; Wilcoxon signed-rank tests were performed as sensitivity analyses, yielding consistent results (Table 1). Effect sizes (Cohen's d for paired samples, calculated as mean difference divided by standard deviation of differences, with 95% confidence intervals) are reported to facilitate interpretation and future sample size calculations. No adjustment for multiple comparisons was applied given the exploratory nature of these analyses; p-values should be interpreted descriptively.

Crossover assessment (*N* = 3): Given the very small sample, results are presented descriptively with individual patient data; no inferential statistics were performed.

Analyses were performed using R (version 4.3).

## Results

### Patient characteristics and electrode placement

**Fig. 1:**
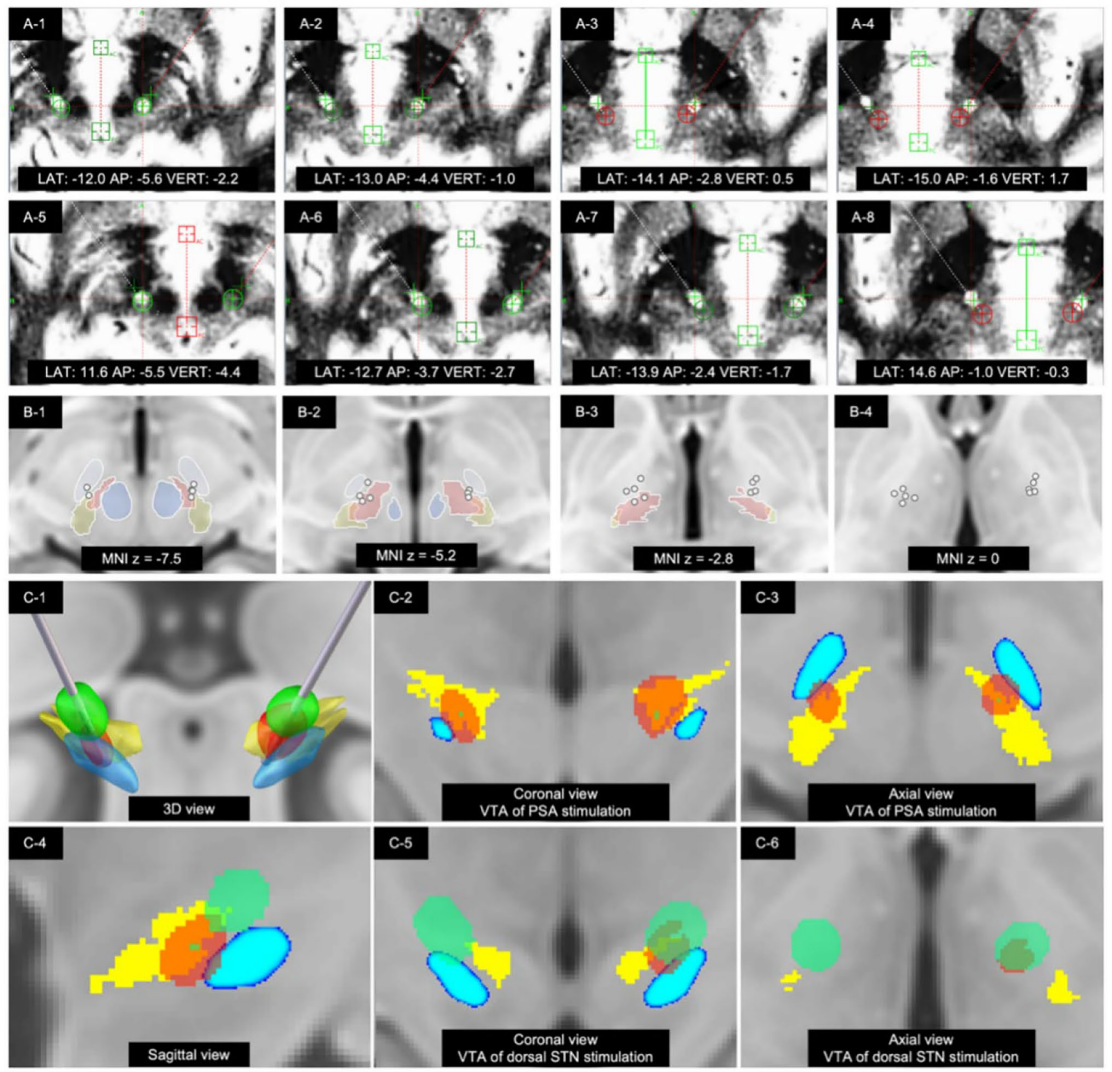
Single-trajectory PSA-STN dual-target DBS. (**A-1**)‒(**A-8**) Representative contact locations shown from the ventral-most to the dorsal-most positions, with AC-PC-based stereotactic coordinates. (**B-1**)‒(**B-4**) Active contact locations displayed in relation to subthalamic area in MNI standard space (blue, red nucleus; light blue, subthalamic nucleus [STN]; rose red, fasciculus cerebellothalamicus [fct]; yellow brown, caudal zona incerta [cZI]). (**C**) A representative volume of tissue activated in relation to the subthalamic area. (**C-1**)‒(**C-6**) A representative VTA shown in 3D and 2D axial views. The ventral stimulation site is referred to as the PSA/cZI-border contact and the dorsal stimulation site as the dorsal STN/Zi-border contact, reflecting relative electrode position along the single trajectory (blue, subthalamic nucleus [STN]; yellow, posterior subthalamic area [PSA]; red, VTA of PSA stimulation; green, VTA of dorsal STN stimulation)

Table 1 presents the demographic and clinical characteristics of the six patients enrolled in this study, comprising four females and two males. The age at surgery ranged from 33 to 65 years. The mean age at disease onset was 46.3 ± 11.7 years, while the mean age at the time of surgery was 57.8 ± 12.4 years. Figure 1 provides an illustration of the pre- and post-operative fused imaging for a representative patient, confirming the final electrode location. The optimized stimulation parameters for each patient at the LFU are detailed in Table 2. The mean duration of symptoms prior to surgery was 143.5 ± 55.4 months.

The mean anterior commissure-posterior commissure-based stereotactic coordinates of the chronically active contacts are detailed in Table 3. The mean coordinates for active PSA contacts were |X|= 10.8 ± 1.1 mm, Y = −4.9 ± 0.9 mm, and Z = −2.4 ± 1.2 mm. The mean coordinates for active STN contacts were |X|= 12.8 ± 1.4 mm, Y = −1.9 ± 1.2 mm, and Z = 1.5 ± 1.5 mm.

**Table 3:** Stereotactic coordinates of active contacts

Patient	Side	PSA			STN		
		X	Y	Z	X	Y	Z
Patient 1	Left	−12.5	−5.0	−1.6	−15.0	−1.6	1.7
	Right	12.1	−4.6	−3.6	14.6	−1	−0.3
Patient 2	Left	−12.1	−6.8	−1.9	−13.7	–4.5	1.8
	Right	11.2	−5.8	−0.2	12.9	−3.4	3.5
Patient 3	Left	‒	‒	‒	−11.8	−2.7	1.6
	Right	‒	‒	‒	13.0	−0.4	2.1
Patient 4	Left	−10.0	−3.9	−2.7	−10.9	−1.8	−0.7
	Right	10.1	−5.2	−4.2	13.7	−2.3	−0.8
Patient 5	Left	−9.7	−3.5	−0.8	−10.7	−1.9	1.5
	Right	9.9	−5.1	−3.2	11.6	−1.8	0.8
Patient 6	Left	−10.9	−4.5	−2.4	−13.5	−1.0	3.8
	Right	9.8	−4.6	−3.5	12.6	−0.2	2.5
Average^b^		10.8 ± 1.1	−4.9 ± 0.9	−2.4 ± 1.2	12.8 ± 1.4	−1.9 ± 1.2	1.5 ± 1.5

The stereotactic coordinates of PSA and STN of the optimal stimulation parameters were shown

*PSA* posterior subthalamic area, *STN* subthalamic nucleus

^a^The midcommissural point was set as origin (0,0,0)

^b^The average of the absolute value of X coordinates was calculated

### Feasibility outcomes

Feasibility was assessed across six predefined objectives. (i) Procedural implementation: All six patients (100%) underwent successful bilateral DBS implantation, with postoperative imaging confirming accurate electrode placement ensuring at least one contact in the PSA and one in the STN for each patient. (ii) Retention and data completeness: All patients (100%) completed their designated follow-up periods (6 months for Patients 1 and 6; 12 months for Patients 2–5) with complete primary outcome data. (iii) Patient-directed programming: All patients successfully managed the three-program switching paradigm at home. (iv) Crossover completion: All three patients assigned to the blinded crossover assessment completed all four stimulation conditions. (v) Patient acceptability: Five of six patients (83.3%) independently selected combined PSA + STN stimulation as their preferred chronic setting. (vi) Safety: The procedure was well-tolerated with no serious adverse events, hardware complications, infections, or intracranial hemorrhages. Transient stimulation-induced paresthesias occurred during programming but resolved with parameter adjustment. One patient (16.7%) experienced mild STN-induced dyskinesia at higher stimulation levels, which was effectively managed through the programming flexibility afforded by the dual-target approach.

### Exploratory clinical efficacy (*N* = 6)

**Fig. 2:**
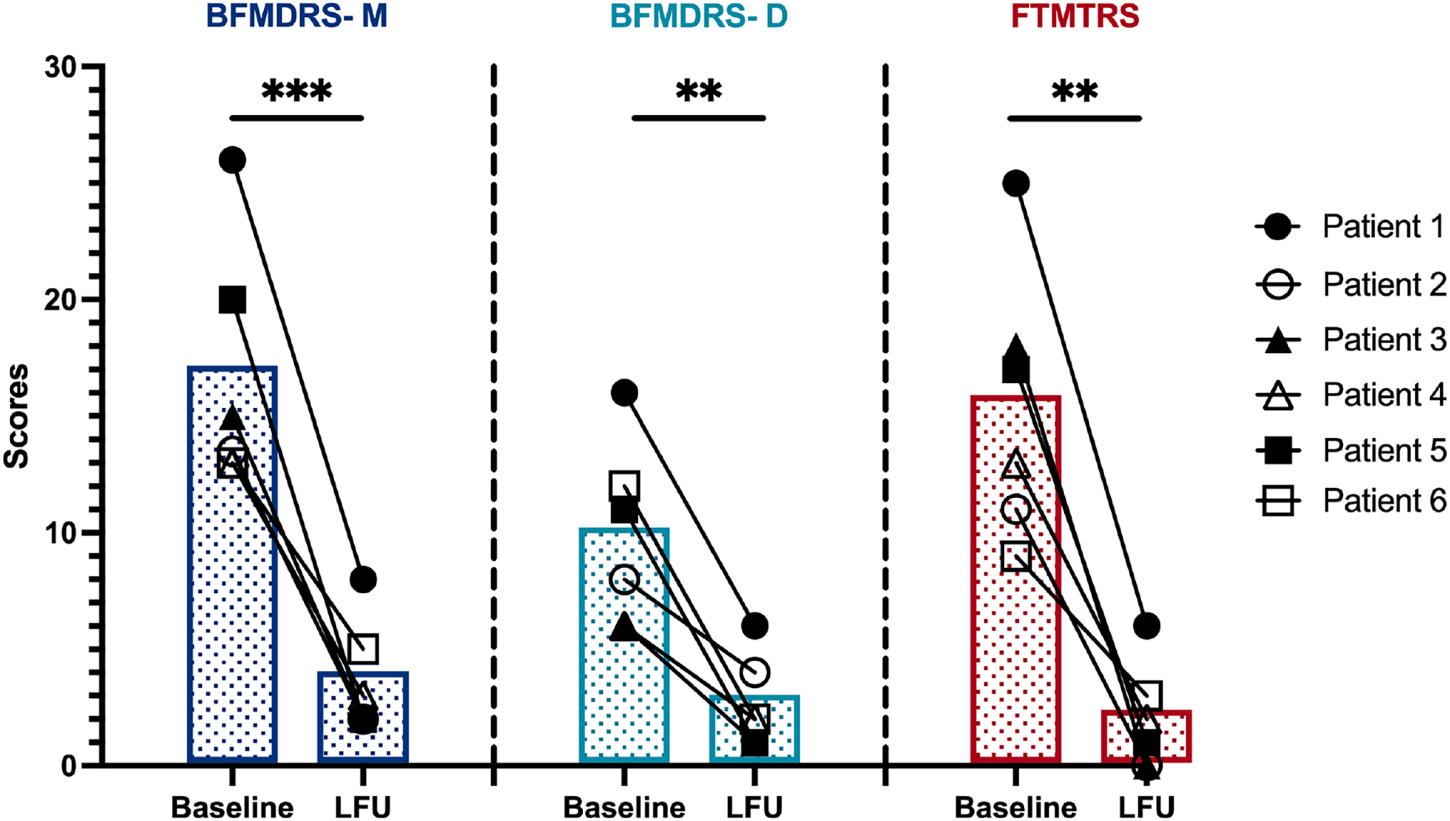
Individual severity of dystonia (BFMDRS-M and BFMDRS-D) and tremor (FTMTRS) before surgery (Baseline) versus the last follow-up (LFU) postoperatively. LFU was 6 months after surgery for Patients 1 and 6, and 12 months for Patients 2, 3, 4, and 5. LFU, last follow-up; BFMDRS-M, BFMDRS movement; BFMDRS-D, BFMDRS disability. The bars represent the mean values for each group. P-values for comparisons between Baseline and LFU are analyzed by parametric tests (Student paired-sample t-tests). A p-value of < 0.05 was considered statistically significant

Exploratory efficacy analyses revealed substantial improvements across all clinical outcomes (Table 1, Fig. 2). The mean BFMDRS-Motor score decreased by 78.1%, from 16.8 ± 5.3 at baseline to 3.7 ± 2.4 at LFU (6 months for Patients 1 and 6; 12 months for Patients 2–5) (Cohen's d = 3.2, 95% CI: 1.8–4.5; *p* = 0.0006). The BFMDRS-Disability score improved by 72.9%, from 9.8 ± 3.9 to 2.7 ± 2.0 (Cohen's d = 2.3, 95% CI: 1.2–3.4; *p* = 0.0025). Tremor showed the most robust improvement, with the mean FTMTRS score decreasing by 87.1%, from 15.5 ± 5.8 to 2.0 ± 2.3 (Cohen's d = 2.7, 95% CI: 1.5–3.9; *p* = 0.0012).

### Comparison of efficacy between stimulation conditions (crossover assessment, *N* = 3)

**Fig. 3:**
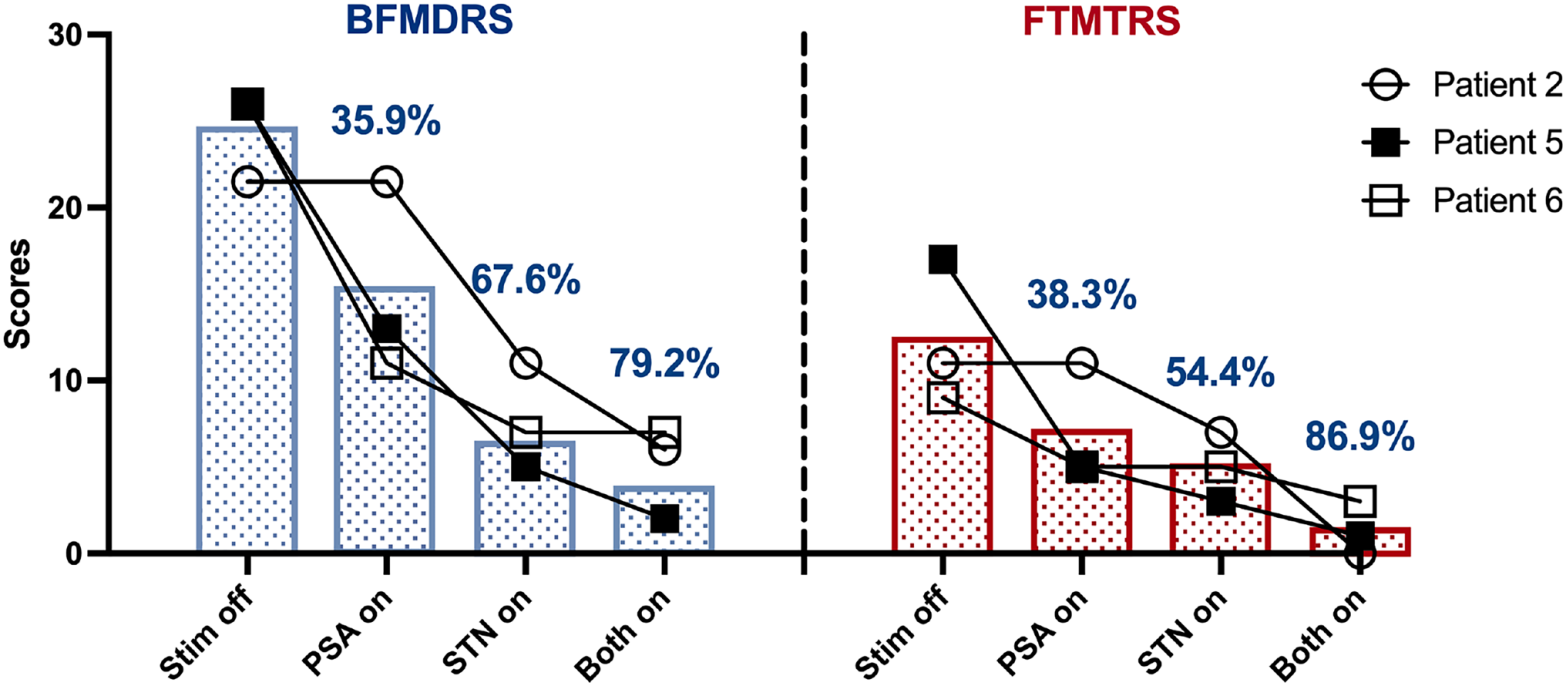
Individual BFMDRS movement and disability scores and FTMTRS scores were evaluated under four conditions: Stim off, PSA on, STN on, and Both on. Bars represent group means, with values above indicating the percentage improvement compared to the Stim off condition

The blinded crossover assessment in three patients (Patients 2, 5, and 6) provided individual-level data on the differential effects of each stimulation condition (Table 4, Fig. 3). Given the very small sample size, results are presented descriptively with emphasis on individual patient responses. The crossover assessment was performed at 12 months post-surgery for Patients 2 and 5, and at 6 months for Patient 6.

**Table 4:** Comparison of efficacy between single−target and dual−target stimulation

Patient	Target	BFMDRS−Motor score	BFMDRS−Disability score	BFMDRS^a^	FTMTRS	Mean improvement, %(BFMDRS)	Mean improvement, %(FTMTRS)
Patient 2	Stim off	13.5	8	21.5	11		
	PSA on	13.5	8	21.5	11	0	0
	STN on	7	4	11	7	48.8	36.4
	Both on	2	4	6	0	72.1	100
Patient 5	Stim off	14	12	26	17		
	PSA on	8	5	13	5	50	70.6
	STN on	2	3	5	3	80.8	82.4
	Both on	1	1	2	1	92.3	94.1
Patient 6	Stim off	14	12	26	9		
	PSA on	8	3	11	5	57.7	44.4
	STN on	5	2	7	5	73.1	44.4
	Both on	5	2	7	3	73.1	66.7
Mean ± SD	Stim off	13.8 ± 0.3	10.7 ± 2.3	24.5 ± 2.6	12.3 ± 4.2		
	PSA on	9.8 ± 3.2	5.3 ± 2.5	15.2 ± 5.6	7.0 ± 3.5	35.9 ± 31.3	38.3 ± 35.7
	STN on	4.7 ± 2.5	3.0 ± 1.0	7.7 ± 3.1	5.0 ± 2.0	67.6 ± 16.7	54.4 ± 24.6
	Both on	2.7 ± 2.1	2.3 ± 1.5	5.0 ± 2.7	1.3 ± 1.5	79.2 ± 11.4	86.9 ± 17.8

^a^BFMDRS=(BFMDRS−Motor score)+(BFMDRS−Disability score)

Individual patient responses: Patient 2 showed no response to PSA-only stimulation (0% improvement in both BFMDRS and FTMTRS), moderate response to STN-only (BFMDRS: 48.8%; FTMTRS: 36.4%), and excellent response to combined stimulation (BFMDRS: 72.1%; FTMTRS: 100%); Patient 5 demonstrated responses across all conditions, with progressive improvement from PSA-only (BFMDRS: 50.0%; FTMTRS: 70.6%) to STN-only (BFMDRS: 80.8%; FTMTRS: 82.4%) to combined stimulation (BFMDRS: 92.3%; FTMTRS: 94.1%); Patient 6 showed similar dystonia improvement with STN-only and combined stimulation (both BFMDRS: 73.1%), but better tremor control with combined stimulation (FTMTRS: 66.7% vs. 44.4% with STN-only).

Across all three patients, combined PSA + STN stimulation consistently provided the best or equivalent symptom control compared to either target alone. The mean BFMDRS improvement was 35.9% ± 29.1% with PSA-only, 67.6% ± 16.7% with STN-only, and 79.2% ± 9.6% with combined stimulation. The mean FTMTRS improvement was 38.3% ± 32.9% with PSA-only, 54.4% ± 21.9% with STN-only, and 86.9% ± 17.0% with combined stimulation.

When comparing the targets, combined stimulation was more effective than STN stimulation alone for improving both dystonia (79.2% vs. 67.6% improvement, respectively) and tremor (86.9% vs. 54.4% improvement, respectively). These individual patterns suggest that while STN stimulation alone provided substantial benefit, the addition of PSA stimulation offered incremental improvement, particularly for tremor control.

### Adverse events

Overall, the surgical procedures were well tolerated in this population. There were no hardware-related side effects, infections, intracranial hemorrhages, or extension or lead fractures from DBS implantation during the follow-up period. Transient, stimulation-induced paresthesia occurred in all six patients during initial programming and subsequent parameter adjustments, but these were resolved by modifying stimulation settings and were not a chronic issue. Specifically, Patient 1 experienced mild STN-induced dyskinesia at higher stimulation levels; this was effectively managed by switching to constant current mode and maintaining a low therapeutic current on that contact. No other common adverse events associated with PSA or STN stimulation were observed at therapeutic settings.

## Discussion

In this pilot study, we present the first clinical evidence on the efficacy and safety of a novel single-lead, dual-target DBS approach for medically refractory dystonic tremor (DT). Our primary finding is that this strategy showed substantial improvements in all six patients, yielding clinically significant improvements in both dystonia (mean 78.1% BFMDRS-Motor improvement) and tremor (mean 87.1% FTMTRS improvement). A key aspect of our study was a patient-directed optimization strategy, which provided strong, real-world validation of the dual-target concept: five of six patients independently selected the combined PSA + STN stimulation program as their preferred setting for chronic therapy, while the sixth achieved excellent symptom control using STN-only stimulation.

A critical question arising from our blinded crossover assessment is why a combined PSA + STN approach is necessary if STN stimulation alone proved more potent than PSA-only stimulation for both dystonia and tremor. The answer lies in two key findings. First, while STN-only was superior to PSA-only, exploratory comparisons in the crossover assessment suggested that combined stimulation of both targets may provide additional benefit over STN-only, indicating a potential synergistic effect rather than simple additivity. Second, this synergy is powerfully illustrated by the real-world experience of Patient 1 (who did not participate in the crossover trial). In her self-directed programming, she found optimal relief only when both targets were active, titrating the PSA-area contact to a high level while requiring the STN-area contact at a lower, but indispensable, electrical current to provide synergistic benefit without inducing dyskinesia. It is particularly noteworthy that this outcome in Patient 1 was achieved using an electrode with a shorter total contact span (7.5 mm vs. 10.5 mm) and narrower inter-contact spacing (0.5 mm vs. 1.5 mm) compared to the rest of the cohort. This observation suggests that the dual-target concept may be feasible across different lead geometries and underscores the importance of precise, individualized surgical targeting and programming.

The pathophysiology of DT is increasingly understood as a "dual-network" disorder, involving pathological oscillations in both the basal ganglia-thalamocortical loop (primarily driving dystonia) and the cerebello-thalamo-cortical (CTC) circuit (primarily driving tremor) [8, 19].Our dual-target approach is therefore well positioned to address this dual pathology. The STN is a critical node in the basal ganglia circuit, and its high-frequency stimulation is thought to work by overriding pathological beta-band oscillations and irregular firing patterns, effectively creating an "informational lesion" that regularizes network output [20, 21]. This modulation is likely the primary driver for the profound dystonia improvement we observed. Concurrently, the PSA is a critical intersection for fibers of the CTC circuit, most notably the dentato-rubro-thalamic tract (DRTT). High-frequency stimulation of the PSA is believed to directly disrupt the propagation of pathological tremor-related oscillations [12]. Therefore, the synergistic effect of our single-lead approach likely stems from its ability to simultaneously modulate these two distinct, yet functionally related, pathological circuits with a single device.

The case of Patient 3, who preferred chronic STN-only stimulation and did not undergo the crossover assessment, also provides valuable insight. While a direct, blinded comparison of conditions for this patient is not possible, his preference may reflect a patient-specific profile where the dystonic symptoms were overwhelmingly dominant, and the profound relief from STN stimulation was sufficient for an excellent outcome. Rather than a limitation of the dual-target hypothesis, this outcome underscores the versatility of the surgical strategy. The ability of a single, well-placed lead to provide robust relief—either through selective stimulation of the STN or combined stimulation of both targets—highlights the platform's powerful adaptability to the significant inter-patient heterogeneity inherent in dystonic tremor.

Stimulation-induced dyskinesia (SID) is a recognized adverse effect of STN-DBS in dystonia, with variable incidence across published series. The relatively low rate observed may relate to the dorsal location of our active contacts (mean z =  + 1.5 mm), as prior studies have associated more ventral/inferior STN stimulation with higher dyskinesia risk [22, 23]. In addition, the single-lead dual-target approach may reduce the required therapeutic load on the STN contact by allowing symptom control to be distributed across PSA and STN stimulation. In Patient 1, SID at higher STN levels was managed by maintaining lower STN current while increasing PSA stimulation, highlighting the practical value of this programming flexibility.

Several anatomical considerations merit clarification. In this study, the terms ‘PSA contact’ and ‘STN contact’ are used to denote ventral and dorsal positions along a single trajectory rather than strict histological boundaries. As illustrated in Fig. 1C, the VTA of the ventral ‘PSA’ contact primarily encompasses the posterior subthalamic area and caudal zona incerta, with partial extension into the ventromedial border of the STN, whereas the VTA of the dorsal ‘STN’ contact primarily involves the dorsal border of the STN and adjacent zona incerta/Forel region. This dorsal subthalamic stimulation site is in line with recent observations [5, 23, 24] that effective stimulation for dystonia often lies at or above the dorsal STN border within the zona incerta-subthalamic region. The inevitable overlap of VTAs across these closely packed structures represents an inherent limitation of conventional omnidirectional DBS electrodes and precludes precise attribution of clinical effects to a single subregion. Future studies employing directional leads and higher-resolution imaging may help to more precisely dissect the respective contributions of PSA/cZI, dorsal STN, and zona incerta modulation.

Several limitations should be acknowledged. This study's primary contribution is demonstrating the feasibility of a novel dual-target DBS approach. While exploratory efficacy improvements were substantial, the retrospective design, small sample size (*N* = 6), and single-center nature preclude definitive efficacy conclusions. The heterogeneity in follow-up timing (6 vs. 12 months), final chronic stimulation programs (combined PSA + STN in 5/6 vs. STN-only in 1/6), and electrode models (SR1200 vs. SR1210) introduces additional variability and may confound cross-patient comparisons in this retrospective series. Genetic testing was not performed; however, given the adult-onset presentation, focal/segmental distribution, and absence of atypical features, the likelihood of identifying a monogenic cause is low. The crossover assessment has specific limitations. The 30 min wash-in/wash-out intervals align with established evidence for rapid tremor responses but are insufficient for complete dystonia assessment; the short-term dystonia improvements observed may reflect acute suppression of phasic dystonic movements. In addition, only three of six patients participated due to personal time constraints or concerns about the extended assessment duration.

Additionally, we did not incorporate formal, time-stamped assessments to quantify the differential onset of improvement for tremor versus dystonia. Literature suggests that tremor responds to PSA/VIM stimulation within seconds [25–28], while dystonia improvement with GPi-DBS typically evolves over weeks to months. However, STN-DBS has been associated with relatively earlier dystonia improvement, with significant benefit observed within the first month [5]. In our cohort, tremor suppression was clinically evident during monopolar screening and the crossover assessment. Short-term reductions in dystonia-related manifestations were also observed in some patients during these evaluations, which may reflect acute suppression of phasic or tremor-coupled dystonic components rather than the full temporal evolution of tonic dystonia. These observations should therefore be interpreted cautiously and considered hypothesis-generating. Future prospective studies should incorporate standardized follow-up intervals, genetic evaluation, objective tremor quantification, extended washout intervals for dystonia assessment, and systematic early assessments to characterize the time course of improvement for each symptom component.

In conclusion, this retrospective pilot study provides strong preliminary evidence that a single-lead, dual-target DBS approach for the PSA and STN is an effective and safe strategy for dystonic tremor. This novel method successfully addresses both dystonic and tremor symptoms and, through a flexible, patient-directed programming paradigm, demonstrates significant potential for truly personalized therapy. While promising, these findings now require validation through larger, multi-center, randomized controlled trials to definitively establish the efficacy of this therapeutic approach.

## Supplementary Information

Below is the link to the electronic supplementary material.

Supplementary file1 (DOCX 15 KB)

## Data Availability

The data that support the findings of this study are available upon reasonable request from the corresponding author.

## References

[1] Albanese A, Bhatia KP, Fung VSC, Hallett M, Jankovic J, Klein C et al (2025) Definition and classification of dystonia. Mov Disord 40(7):1248–1259. 10.1002/mds.30220 40326714 10.1002/mds.30220 PMC12273609

[2] Fasano A, Bove F, Lang AE (2013) The treatment of dystonic tremor: a systematic review. J Neurol Neurosurg Psychiatry 85(7):759–769. 10.1136/jnnp-2013-305532 24167042 10.1136/jnnp-2013-305532

[3] Gironell A, Kulisevsky J (2009) Diagnosis and management of essential tremor and dystonic tremor. Ther Adv Neurol Disord 2(4):215–222. 10.1177/1756285609104791 21179530 10.1177/1756285609104791 PMC3002636

[4] Tsuboi T, Au KLK, Deeb W, Almeida L, Foote KD, Okun MS et al (2020) Motor outcomes and adverse effects of deep brain stimulation for dystonic tremor: a systematic review. Parkinsonism Relat Disord 76:32–41. 10.1016/j.parkreldis.2020.06.008 32559631 10.1016/j.parkreldis.2020.06.008

[5] Lin S, Shu Y, Zhang C, Wang L, Huang P, Pan Y et al (2023) Globus pallidus internus versus subthalamic nucleus deep brain stimulation for isolated dystonia: a 3-year follow-up. Eur J Neurol 30(9):2629–2640. 10.1111/ene.15895 37235703 10.1111/ene.15895

[6] Lin S, Wu Y, Li H, Zhang C, Wang T, Pan Y et al (2019) Deep brain stimulation of the globus pallidus internus versus the subthalamic nucleus in isolated dystonia. J Neurosurg 132(3):721–732. 10.3171/2018.12.JNS181927 30849756 10.3171/2018.12.JNS181927

[7] Hedera P, Phibbs FT, Dolhun R, Charles PD, Konrad PE, Neimat JS et al (2013) Surgical targets for dystonic tremor: considerations between the globus pallidus and ventral intermediate thalamic nucleus. Parkinsonism Relat Disord 19(7):684–686. 10.1016/j.parkreldis.2013.03.010 23611688 10.1016/j.parkreldis.2013.03.010

[8] Trompette C, Giordana C, Leplus A, Grabli D, Hubsch C, Marsé C et al (2022) Combined thalamic and pallidal deep brain stimulation for dystonic tremor. Parkinsonism Relat Disord 103:29–33. 10.1016/j.parkreldis.2022.08.003 36029608 10.1016/j.parkreldis.2022.08.003

[9] Paoli D, Mills R, Brechany U, Pavese N, Nicholson C (2023) DBS in tremor with dystonia: VIM, GPi or both? A review of the literature and considerations from a single-center experience. J Neurol 270(4):2217–2229. 10.1007/s00415-023-11569-6 36680569 10.1007/s00415-023-11569-6 PMC10025201

[10] Blomstedt P, Sandvik U, Fytagoridis A, Tisch S (2009) The posterior subthalamic area in the treatment of movement disorders: past, present, and future. Neurosurgery. 10.1227/01.NEU.0000345643.69486.BC 19487881 10.1227/01.NEU.0000345643.69486.BC

[11] Kvernmo N, Konglund AE, Reich MM, Roothans J, Pripp AH, Dietrichs E et al (2022) Deep brain stimulation for arm tremor: a randomized trial comparing two targets. Ann Neurol 91(5):585–601. 10.1002/ana.26317 35148020 10.1002/ana.26317 PMC9311445

[12] Barbe MT, Reker P, Hamacher S, Franklin J, Kraus D, Dembek TA et al (2018) DBS of the PSA and the VIM in essential tremor: a randomized, double-blind, crossover trial. Neurology 91(6):e543–e550. 10.1212/WNL.0000000000005956 29970404 10.1212/WNL.0000000000005956

[13] Sun X, Shen R, Lin Z, Wang T, Wang L, Huang P et al (2024) Optimizing deep brain stimulation in essential tremor: a randomized controlled trial for target consideration. Neurosurgery 95(1):63–75. 10.1227/neu.0000000000002839 38270451 10.1227/neu.0000000000002839 PMC11155559

[14] Thabane L, Ma J, Chu R, Cheng J, Ismaila A, Rios LP et al (2010) A tutorial on pilot studies: the what, why and how. BMC Med Res Methodol 10:1. 10.1186/1471-2288-10-1 20053272 10.1186/1471-2288-10-1 PMC2824145

[15] Eldridge SM, Lancaster GA, Campbell MJ, Thabane L, Hopewell S, Coleman CL et al (2016) Defining feasibility and pilot studies in preparation for randomised controlled trials: development of a conceptual framework. PLoS ONE 11(3):e0150205. 10.1371/journal.pone.0150205 26978655 10.1371/journal.pone.0150205 PMC4792418

[16] Lin Z, Huang P, Zeng Z, Zhang C, Tan Y, Li D (2024) Single-trajectory deep brain stimulation of the posterior subthalamic area and subthalamic nucleus for dopamine-resistant parkinsonian tremor: a case report. Deep Brain Stimul 4:42–46. 10.1016/j.jdbs.2024.01.001

[17] Lancaster GA, Dodd S, Williamson PR (2004) Design and analysis of pilot studies: recommendations for good practice. J Eval Clin Pract 10(2):307–312 15189396 10.1111/j..2002.384.doc.x

[18] Cohen J (1988) Statistical power analysis for the behavioral sciences. 2nd ed. Lawrence Erlbaum Associates, Hillsdale (NJ)

[19] Tsuboi T, Wong JK, Eisinger RS, Okromelidze L, Burns MR, Ramirez-Zamora A et al (2021) Comparative connectivity correlates of dystonic and essential tremor deep brain stimulation. Brain 144(6):1774–1786. 10.1093/brain/awab074 33889943 10.1093/brain/awab074

[20] Wong JK, Viswanathan VT, Nozile-Firth KS, Eisinger RS, Leone EL, Desai AM et al (2020) STN versus GPi deep brain stimulation for action and rest tremor in Parkinson’s disease. Front Hum Neurosci 14:578615. 10.3389/fnhum.2020.578615 33192410 10.3389/fnhum.2020.578615 PMC7651783

[21] Zimnik AJ, Nora GJ, Desmurget M, Turner RS (2015) Movement-related discharge in the macaque globus pallidus during high-frequency stimulation of the subthalamic nucleus. J Neurosci 35(9):3978–3989. 10.1523/JNEUROSCI.4899-14.2015 25740526 10.1523/JNEUROSCI.4899-14.2015 PMC4348192

[22] Wang N, Wang K, Wang Q, Fan S, Fu Z, Zhang F et al (2020) Stimulation-induced dyskinesia after subthalamic nucleus deep brain stimulation in patients with meige syndrome. Neuromodulation 24(2):286–292. 10.1111/ner.13284 32964635 10.1111/ner.13284

[23] Yin F, Zhao M, Yan X, Li T, Chen H, Li J et al (2022) Bilateral subthalamic nucleus deep brain stimulation for refractory isolated cervical dystonia. Sci Rep 12(1):7678. 10.1038/s41598-022-11841-1 35538160 10.1038/s41598-022-11841-1 PMC9090754

[24] Butenko K, Neudorfer C, Dembek TA, Hollunder B, Meyer GM, Li N et al (2025) Engaging dystonia networks with subthalamic stimulation. Proc Natl Acad Sci U S A 122(2):e2417617122. 10.1073/pnas.2417617122 39773021 10.1073/pnas.2417617122 PMC11745339

[25] Chung M, Huh R (2015) Different clinical course of pallidal deep brain stimulation for phasic- and tonic-type cervical dystonia. Acta Neurochir (Wien). 10.1007/s00701-015-2646-7 26611690 10.1007/s00701-015-2646-7

[26] Koeglsperger T, Palleis C, Hell F, Mehrkens JH, Bötzel K (2019) Deep brain stimulation programming for movement disorders: current concepts and evidence-based strategies. Front Neurol 10:410. 10.3389/fneur.2019.00410 31231293 10.3389/fneur.2019.00410 PMC6558426

[27] Perera T, Yohanandan SAC, Vogel AP, McKay CM, Jones M, Peppard R et al (2015) Deep brain stimulation wash-in and wash-out times for tremor and speech. Brain Stimul. 10.1016/j.brs.2015.01.156

[28] Butler RD, Brinda AK, Blumenfeld M, Bryants MN, Grund PM, Pandey SR et al (2024) Differentiating postural and kinetic tremor responses to deep brain stimulation in essential tremor. Mov Disord Clin Pract 12(2):166–176. 10.1002/mdc3.14256 39508598 10.1002/mdc3.14256 PMC11802662

