# Supplementary material for: Efficacy and safety of single-trajectory posterior subthalamic area and subthalamic nucleus deep brain stimulation for dystonic tremor: a retrospective pilot study
Source: J Neurol. 2026 Jan 6;273(1):60. doi: 10.1007/s00415-025-13569-0 (PMC12775118; doi:10.1007/s00415-025-13569-0)
Supplement: Supplementary file 1 — Supplementary file1 (DOCX 15 KB) [file 415_2025_13569_MOESM1_ESM.docx]

**Table S1. The Leksell stereotactic frame arc and ring angles for all trajectories**

| Patient | side | Coronal angle | Sagittal angle |
| --- | --- | --- | --- |
|  |  |  |  |
|  |  |  |  |
| Patient 1 | left | 112 | 44 |
|  | Right | 67 | 40 |
| Patient 2 | left | 109 | 64 |
|  | Right | 70 | 60 |
| Patient 3 | left | 111 | 72 |
|  | Right | 68 | 72 |
| Patient 4 | left | 109 | 44 |
|  | Right | 69 | 46 |
| Patient 5 | left | 107 | 54 |
|  | Right | 75 | 54 |
| Patient 6 | left | 109 | 60 |
|  | Right | 71 | 56 |
